# The influence of body size and net diversification rate on molecular evolution during the radiation of animal phyla

**DOI:** 10.1186/1471-2148-7-95

**Published:** 2007-06-26

**Authors:** Eric Fontanillas, John J Welch, Jessica A Thomas, Lindell Bromham

**Affiliations:** 1 Centre for the Study of Evolution, School of Life Sciences, University of Sussex, Falmer, Brighton, BN1 9QG, UK; 2 Institute of Evolutionary Biology; School of Biological Sciences; University of Edinburgh, West Mains Rd., Edinburgh, EH9 3JT, UK; 3 Centre for Macroevolution and Macroecology, School of Botany and Zoology, Australian National University, Canberra, A.C.T. 0200 Australia

## Abstract

**Background:**

Molecular clock dates, which place the origin of animal phyla deep in the Precambrian, have been used to reject the hypothesis of a rapid evolutionary radiation of animal phyla supported by the fossil record. One possible explanation of the discrepancy is the potential for fast substitution rates early in the metazoan radiation. However, concerted rate variation, occurring simultaneously in multiple lineages, cannot be detected by "clock tests", and so another way to explore such variation is to look for correlated changes between rates and other biological factors. Here we investigate two possible causes of fast early rates: change in average body size or diversification rate of deep metazoan lineages.

**Results:**

For nine genes for phylogenetically independent comparisons between 50 metazoan phyla, orders, and classes, we find a significant correlation between average body size and rate of molecular evolution of mitochondrial genes. The data also indicate that diversification rate may have a positive effect on rates of mitochondrial molecular evolution.

**Conclusion:**

If average body sizes were significantly smaller in the early history of the Metazoa, and if rates of diversification were much higher, then it is possible that mitochondrial genes have undergone a slow-down in evolutionary rate, which could affect date estimates made from these genes.

## Background

Dating the origin of the animal kingdom (Metazoa) has been a long standing challenge in evolutionary biology, and has important implications for our understanding of macroevolutionary processes and the tempo and mode of evolution [1,2]. One widely held view, the "Cambrian explosion" hypothesis, is that the major groups of animals diverged near-simultaneously during (or just before) the early Cambrian period [3]. This hypothesis stems from the sudden appearance of many metazoan phyla in the fossil record in the early Cambrian (from 542 to 488 million years ago, Mya). For while some consider the Ediacaran fauna (approximately 600 to 542 Mya, however, most Ediacaran organisms are between 565 and 541 million years old [4]) to be ancestral metazoans (e.g., [5,6]) and putative Precambrian bilaterian fossils have been reported (e.g., refs. [7-10]), there are as yet no undisputed bilaterian fossils in the Proterozoic [11] and the several hypotheses that support the existence of Precambrian bilaterians all seem to have some weaknesses (for a review, see [12]). The apparently rapid evolution of the major groups of animals in the early Cambrian has long been considered a challenge to the universal application of Darwinian gradualism [13].

Against this "Cambrian explosion" hypothesis, it has been argued that limitations of the fossil record may have obscured the earlier evolutionary history of the Metazoa [2,14], and it therefore remains possible that the metazoan phyla arose not by an explosive radiation, but by a long period of diversification [15]. Arguments for a Precambrian diversification of animal phyla have come from many sources, including phylogenetic [16] and palaeoecological analyses [17], but one of the most consistent lines of evidence has come from DNA sequence data. Molecular clock studies have traditionally relied upon the assumption that rates of molecular evolution are roughly constant over time and between lineages, meaning that genetic distance can be converted into an estimate of time since divergence. Since the pioneering work of Runnegar [18], many studies have used the assumption of constant molecular rates to date metazoan origins. These studies have produced a wide range of date estimates for the origin of bilaterians, with the date estimates for the split between the protostomes and deuterostomes ranging from 630 Mya [19] to 1200 Mya ([20]). Although all strict molecular clock studies (i.e. based on a constant molecular rate) have placed the origin of bilaterians before the first undisputed fossil evidence in the Cambrian, these studies have been criticized for many reasons, particularly because evidence suggests that rates of molecular evolution vary widely between different animal lineages (e.g.,[21]).

In response to these criticisms, molecular dating methods have now been developed that allow for variation in rate of molecular evolution (for reviews see [22,23]). While most rate-variable date estimates for the origins of major animal lineages are much older than the earliest bilaterian fossils (e.g. [24]), these methods have also produced some dates that are considered more compatible with the fossil evidence (Peterson *et al*. date the last common bilaterian ancestor between 573 and 656 Mya, [25]; Aris-Brosou and Yang date the deuterostome/protostome split on average 582 ± 112 Mya, [26]). However, these very young molecular estimates rely on procedures expected on external grounds to yield artefactually young dates. For example, some procedures include methods of branch length estimation that are known to yield underestimates such as parsimony or minimum evolution methods [27], maximum likelihood models that neglect between-site rate heterogeneity [28], or use Bayesian methods with strong prior assumptions biased towards producing young date estimates and fast early molecular rates [26,29,30]. Therefore, the conflict between molecular and palaeontological dates for the origin of the animal phyla remains unresolved (for a review of this debate see [12]).

One possible explanation for the disagreement between palaeontological and molecular estimates of animal origins would be if rates of molecular evolution were faster in all or most bilaterian lineages at the base of Cambrian [21,26]. But at this time, there is no molecular evidence for higher rates of molecular evolution in the Cambrian. One possible reason for this, is that such concerted changes in rate (e.g. where many lineages increase in rate at the same time) are a particular problem in molecular dating studies. Although new dating methods allow autocorrelation between ancestral and descendant lineages, they make no allowance for autocorrelation of rates between descendant lineages. Moreover, such autocorrelated changes in rate between lineages cannot be identified by "clock tests" which aim to identify departures from rate constancy [31]. Because concerted changes in rate cannot be detected from branch length estimates, rate-variable molecular dating methods will only account for such changes if they are incorporated into the estimation as prior knowledge.

All molecular dating methods must specify some form of prior assumptions about both divergence dates and molecular rates. Variable-rate methods can only accommodate concerted changes in rate in two ways: through the date prior or the rate prior. Rate-variable methods may specify a date prior that allows a concerted rate change by compressing or stretching all branches in part of the phylogeny. This can be achieved in one of two ways [22]: using a model (such as a birth-death process) that stretches portions of the tree, or via multiple calibrations either side of the rate change. In practise, most rate-variable dating methods have taken these approaches. However, the use of evolutionary models to constrain rate-variable dates is problematic in that the assumptions embodied in such methods (for example, birth and death model of speciation), often do not adequately reflect the biological systems being modelled. This is particularly worrisome for some studies of the metazoan radiation, where it can be shown that the assumptions of the models are the main determinant for the young date estimates [30]. The use of multiple calibrations as constraints on the nodes of interest has the advantage of using empirical data to constrain solutions. However it makes the rate-variable date estimates highly dependent on external calibrations, and prevents their use as a source of temporal information that is independent of the fossil record (which is important for testing hypotheses such as the Cambrian explosion).

Alternatively, variable-rate methods can specify a rate prior that favours concerted changes. Again, this could be done in one of two ways: by simply specifying a directionally biased model or by including additional information that has been independently found to be associated with a change in rates (e.g. a body size effect on molecular rates). Some rate-variable methods have included directionally-biased models (e.g. [26]), in which case the young date estimates are a result of the assumption of fast early rates, not a test of this hypothesis. The use of independent rate priors to take into account concerted change in rate-variable molecular dating has thus far never been applied, and would rely upon identifying correlates of molecular rate that may have varied systematically across the phylogeny [22,32]. For example, if a life history trait was found that correlated negatively with the rate of molecular evolution, and if that trait could be shown to have increased in most metazoan lineages since the Cambrian, then this might imply that rates were generally faster in Cambrian. Faster rates in the Cambrian would produce a consistent bias in molecular date estimates, making molecular dates systematically overestimated whatever method or data was used. This is the motivation for the present study. In this paper, we investigate two potential correlates of molecular rate variation that may, in this way, help to explain the discrepancy between molecular and fossil dates of the metazoan radiation.

The first potential correlate of molecular rate is body size. It has been proposed that ancestral small size in metazoan lineages would have increased rates of molecular evolution (see discussion in [21]). This theory is supported by interpretations of the fossil record suggesting that the earliest metazoans were much smaller than extant species, perhaps resembling modern meiofauna [33] or ciliated metazoan larvae [34]. However alternative hypotheses suggest the early presence of complex and large-bodied metazoans [12]. The potential correlation between ancestral small size in metazoan and fast early rates is also supported by the observation of a negative relationship between body size and rate of molecular evolution in several vertebrates clades, including birds [35], reptiles [36] and mammals [37]. However, a recent study of invertebrates which used sequence data from 330 species from five different phyla, found that, while rates of molecular evolution varied significantly, there was no evidence that rate scaled with body size [38]. However that analysis was restricted to comparisons between species, genera and families. It is possible that an influence of body size on rates of molecular evolution in the early stages of animal evolution will only be evident when extremely divergent lineages are compared. It is therefore interesting to investigate whether body size is associated with rate of molecular evolution at higher taxonomic levels (e.g. phylum, class and order).

We also investigate a second potential correlate of variation in rate of molecular evolution: net diversification rate (i.e. speciation rate minus extinction rate). The Cambrian explosion hypothesis suggests that net diversification rate may have been greatly inflated during the early metazoan radiation [39]. A correlation between net diversification rate and substitution rate has been observed in flowering plants [40], and for a collection of 56 phylogenies including several phylogenies of metazoans [41]. Theory also suggests that several speciation modes are expected to result in an increase in substitution rate (see Discussion). Here we use estimates of extant species number to represent the net diversification rate for the metazoan taxa.

In this study we use a phylogenetic comparative approach to compare rates of molecular evolution between metazoan phyla, classes, orders and families [see Additional file 1] that differ in taxon average body size and in species number. We examine rates of molecular evolution in nine different genes (including mitochondrial and nuclear, protein- and RNA-coding genes). Our study shows strong evidence for a negative correlation between substitution rate and body size for the mitochondrial genes. There is also some evidence of a positive correlation between substitution rate and net diversification rate for the mitochondrial genes. This implies that if the earliest metazoans were much smaller than their modern descendants, and/or if the net diversification rate was significantly higher, then it is possible that rates of mitochondrial molecular evolution could have been higher during the early Cambrian. If this is true, then molecular date estimates based on these sequences could systematically overestimate the date of origin of metazoan phyla.

## Results

### The relationship between body size and molecular rate in mitochondrial genes

The mitochondrial sequences show clear evidence of a negative association between body size and rate of molecular evolution (Table 1). The C20 concatenated alignment shows a significant negative correlation under the two partitions of the sequence data (Table 1; C20 shows also a significant negative correlation under the "gene partitions model" implemented in PAML, results not shown). This observation is also supported at the individual gene level, with two of the six mitochondrial genes showing significant correlations at the 5% level under the "No partition" model (COII and NADH4), and four of the six showing significant correlations at the 10% level under the "Codon partitions" model (COII, COIII, cytB, NADH4). Furthermore, correlation coefficients for all alignments were negative, whether significant or not. This negative relationship is also evident from scatterplots of the data [see Additional file 2].

**Table 1:** Results of the Spearman's rank correlation tests. The outputs of the Spearman's rank correlation test (correlation coefficient r_s _and p-values) are used to explore the relationship between the rate of molecular evolution and the biological variables under study (body size and species number). Results are presented for nuclear genes, mitochondrial genes and concatenation of mitochondrial genes. Concatenations of mitochondrial genes are given for the complete set of taxa (C100), and a set excluding pairs of taxa that contained more than 20% of missing sequence (C20). Significant correlations at the 5% level are shown in bold.

		**Molecular rate vs body size**		**Molecular rate vs species number**	
		
		**r_s _(p-value)**		**r_s _(p-value)**	
		
**Genes**		**No partition**	**Codon positions partitions**	**No partition**	**Codon positions partitions**
**Nuclear**	**18S**	-0.05 (0.807)	-	-0.01 (0.969)	-
	**28S**	0.08 (0.745)	-	0.15 (0.535)	-
	**ef1a**	-0.39 (0.165)	-0.42 (0.141)	-0.04 (0.905)	0.04 (0.886)
**Mitochondrial**	**COI**	-0.12 (0.617)	-0.19 (0.422)	0.07 (0.776)	0.22 (0.345)
	**COII**	**-0.55 (0.025)**	**-0.50 (0.043)**	0.33 (0.201)	0.28 (0.272)
	**COIII**	-0.35 (0.174)	-0.44 (0.082)	0.52 (0.031)	**0.49 (0.047)**
	**cytB**	-0.42 (0.106)	-0.48 (0.060)	0.42 (0.103)	0.32 (0.221)
	**NADH1**	-0.35 (0.165)	-0.34 (0.184)	0.29 (0.260)	0.36 (0.153)
	**NADH4**	**-0.61 (0.012)**	-0.45 (0.074)	0.06 (0.815)	0.05 (0.863)
**Concatenation**	**C100**	-0.32 (0.175)	-0.29 (0.214)	0.22 (0.341)	0.16 (0.497)
	**C20**	**-0.53 (0.037)**	**-0.51 (0.046)**	0.39 (0.132)	0.35 (0.187)

### The relationship between net diversification rate and molecular rate in mitochondrial genes

There is also evidence, albeit weaker, that the rate of molecular evolution in mitochondrial genes is positively correlated with clade size, which in this study is used to represent net diversification rate. While this relationship is only significant for a single gene (COIII: |rs| = 0.52; p-value = 0.031), the coefficients are positive in all cases, and the scatterplots confirm the positive trend [see Additional file 3]. It is interesting to note the tendency of the mitochondrial genes to produce "polygonal plots" as when the difference in species number is less than a factor of two (i.e of 1 on our log_2 _scale), then the rate difference is highly variable, whereas the relationship appears somewhat more obvious when the richness differences are more dramatic. If this variation in rate contrast associated with small differences in species number is due to a measurement error effect (i.e. error in estimation of species number is expected to have a bigger impact on the small contrasts), it could add noise to the observed correlation and hide a stronger positive correlation between diversification rate and molecular rate.

### Partial correlation test between molecular rate, body size and net diversification rate in mitochondrial genes

Because the negative correlation observed between molecular rate and body size could be an indirect result of a relationship involving the net diversification rate, we performed partial correlation tests which did not indicate any evidence for any relationship involving molecular rate, body size and net diversification rate together. However, the interpretation of these results is problematic for data which departs from a normal distribution [42]. Moreover, consistent with the study by Orme *et al*. [43] our data indicate no evidence for a relationship between body size and net diversification rate (p-value~1, results not shown); the inclusion of the corresponding non-significant correlation coefficient in the partial correlation tests makes the interpretation of the results of such tests difficult.

### Study of correlates in nuclear genes

In contrast to the mitochondrial genes, the nuclear genes do not show any significant relationship between molecular rate and either body size or net diversification rate. The correlation coefficients vary in sign, and are often close to zero (Table 1). The sole exception is the correlation between body size and substitution rate for ef1a which, though non-significant, is close in magnitude to those observed for the mitochondrial sequences.

## Discussion

This study provides evidence that the rate of molecular evolution in mitochondrial genes is negatively correlated with body size for deep comparisons within the Metazoa. We have confidence in this result for several reasons. First, significant results were obtained for both concatenated and single-gene sequences, despite the use of relatively conservative non-parametric statistics. Second, all trends were in the same direction (negatively correlated) whether or not tests were significant (Table 1). Third, our results are not confounded by evolutionary relationships between sequences (a common problem in the study of rates of molecular evolution), because we used phylogenetically independent comparisons, chosen from a phylogeny supported by several recent studies [24,44-47].

**Figure 1:**
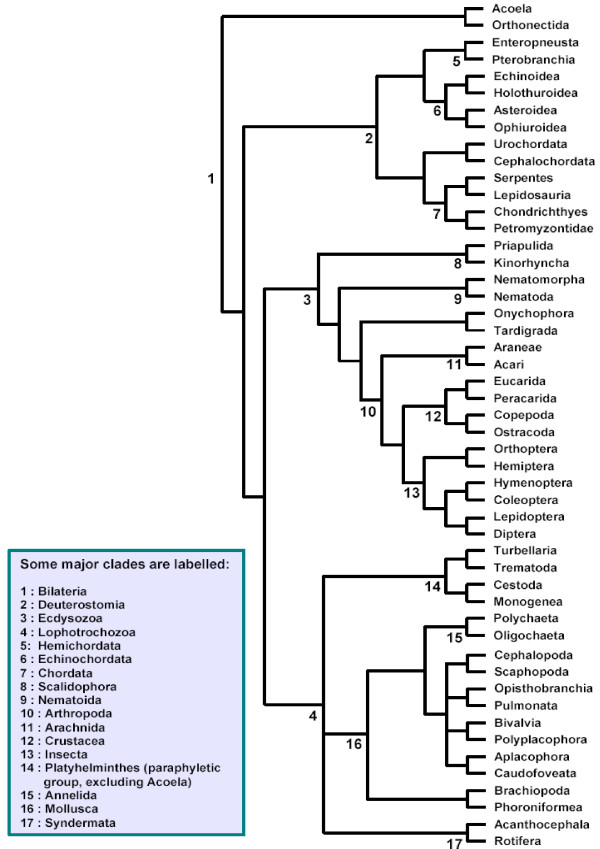
Assumed metazoan phylogeny based on multiple sources (see methods).

This result is in accordance with previous studies of rate of molecular evolution in vertebrates [37]. However, it is in contrast to that of Thomas *et al*. [38], which found no evidence of any body size effect over a wide range of invertebrate taxa. So why does this study find evidence of a relationship in invertebrates, when the previous study did not? The majority of data points in this study are comparisons between invertebrate taxa (see Figure 1), so the association is unlikely to be simply a result of the inclusion of vertebrate comparisons (which were not included in Thomas *et al*. study). Furthermore, the two studies employ very similar methodologies, comparable sequence data, and overlapping taxonomic coverage. Importantly, though, the comparisons in Thomas *et al*. are at a different level of the metazoan phylogeny to those used in the present study. Indeed, this study was designed to extend that of Thomas *et al*., by sampling a part of the metazoan phylogeny not included in the earlier study. This is an important distinction, because the deeper comparisons included in this study (phylum, order and class) are potentially more relevant to the problem of molecular dates for the metazoan radiation than the shallower comparisons made by Thomas *et al*. (species, genus and family).

There are several possible reasons why this difference in comparison depth may be responsible for the different results obtained by the two studies. The use of much deeper comparisons may have increased the power of the test in three ways. Firstly, the comparison pairs used here have longer branch lengths. This means that every comparison samples more substitutions, which will have increased the accuracy of the rate estimates (as long as the data are not saturated). Secondly, the body size differences were typically much greater in the present work: the comparisons used by Thomas *et al*. differed in body size by a ratio of less than 8:1, whereas our comparisons have an average body size ratio of 125:1 [see Additional file 4]. Thirdly, deeper comparisons may have greater resolving power if they overcome confounding effects of unknown variables, which may fluctuate over evolutionary time. For example, a hidden causal variable might have masked the effects of body size in shallow comparisons, if such a variable was fluctuating on timescales comparable to the divergence times of species or genera.

In addition to differences in resolving power, it is possible that the discrepancy between the two studies is a result of sampling different parts of metazoan diversity, and that rates of molecular evolution really were affected by body size in the lineages included in the present study, but not in those sampled by Thomas *et al*. [38]. One possible difference between the datasets is that most of the phylogenies analysed by Thomas *et al*. contained exclusively terrestrial invertebrates while most of the substitutions measured in the current study will have taken place in a marine environment (even for phyla with many living terrestrial representatives). It has been suggested that marine organisms may have consistently different rates of molecular evolution due to, for example, larger effective population sizes [48] or a direct effect of salt concentration on mutation rates [49].

A further possibility, and one that is relevant to the issue of dating the metazoan radiation, is that the association between body size and rates was more pronounced in the early period of metazoan evolution. In this case, the effect detected in this study might not be detected by studies of more recent metazoan lineages. However, we know of no reasons for such a temporal shift in the tempo and mode of molecular evolution.

Providing plausible causal explanations for the body size effect we have observed is greatly complicated by the fact that so many life history traits, and other putative causal variables, tend to covary with body size [37,50]. In particular, two variables that co-vary with body size have been put forward as potential causes of variation in molecular rates. Generation time may influence rates of molecular evolution because organisms that copy their germline DNA more often per unit time are expected to incur more DNA replication errors, which could increase the mutation rate. [35,50]. Alternatively, metabolic rate may influence rates of DNA damage, through the production of reactive oxygen species (ROS) which are by-products of metabolism [51]. Indeed, it has been suggested that metabolic rate is the primary driver of rates of molecular evolution [50], linked to body mass through allometric scaling, perhaps through a "3/4 power law" (for a review, see [52]). However, the only studies that have explicitly compared these two variables found no effect of metabolic rate on molecular rates above its covariation with generation time and body size [35,37]. Because both metabolic rate and generation time may scale with body size for many metazoan lineages [52-55], we are unable to test which provides the better explanation for the pattern we observe. However, it is possible that the metabolic rate effect may provide an explanation for our observation of a body size effect in mitochondrial genes but not in nuclear genes. Because mitochondria are the site of production of ROS, it is possible that DNA damage from metabolites accounts for a greater proportion of mutations in mitochondrial DNA than in nuclear DNA, potentially explaining why we observe an effect in the mitochondrial genes alone. Alternatively, it is possible that metabolic rate might influence the rate of adaptation of mitochondrial genes, thus potentially affecting the substitution rate [56]. However, a recent study found no association between metabolic rate and rate of molecular evolution for a large metazoan dataset [57].

Alternatively, the failure to detect a body size pattern in rates for nuclear genes may simply be an artefact of relative lack of power: the nuclear sequences typically contained fewer substitutions per branch than did the mitochondrial sequences, reducing our ability to accurately measure the substitution rate, and so reducing the power of the tests. It is also possible that the effect we have detected applies chiefly to synonymous substitutions in protein coding sequences. Such substitutions will dominate estimates of the overall rate in mitochondrial, but not in nuclear genes, because the former tend to have a much lower ratio of amino-acid-changing to synonymous substitutions [56,58]. In support of this suggestion is the observation that, in mammals, synonymous substitutions show more evidence of lineage-specific rate variation than do amino-acid-changing substitutions [59].

While the existence of a body size effect for deep metazoan comparisons is indicated relatively clearly by our analysis, evidence for a species number effect is more equivocal. Although a significant result was obtained only for a single mitochondrial gene (COIII), the agreement of the signs of the correlations across all of the mitochondrial genes, together with observation of the scatterplots [see Additional file 3], tentatively suggest that rates of mitochondrial evolution may increase with net diversification rate. These patterns are consistent with previous empirical work that has found a correlation between diversification rate and rate of molecular evolution [40,41]. Net diversification rate is the result of addition of lineages by speciation and removal of lineages by extinction. It is difficult to predict the influence of variation in extinction rates on rates of molecular evolution, however some theoretical models do predict a link between speciation and rate of molecular change, and it is possible that this is the underlying cause of the relationship. For example, the population splitting involved in speciation implies reduced effective population size (*N*_*e*_) which could increase the fraction of mutations that are effectively neutral (with 0 <*N*_*e*_*|s*| < < 1) and therefore able to reach fixation. (e.g., [60]). Speciation involving a founder event [61] may involve an even more drastic reduction in *N*_*e*_. Alternatively, speciation involving adaptation to a new niche, or runaway sexual selection, may generate adaptive substitutions which could cause a detectable increase in substitution rate. However, in contrast to the genome-wide effects of a reduction in *N*_*e*_, such adaptive scenarios would be likely to generate gene-specific patterns which are unlikely to have been detected by our study, particularly because we have targeted "house-keeping genes" involved in basic cellular processes common to all organisms (i.e. ribosomal RNAs and oxidative-chain proteins). Another consideration is that divergence itself can lead to speciation due to the accumulation of hybrid incompatibilities (an idea developed in the Bateson-Dobzhansky-Muller model; [62,63]), and this applies regardless of how the substitutions are caused (i.e. whether they are drift-mediated or adaptive substitutions). While these direct causal explanations are certainly possible, as with the body size effect, it is also possible that we are observing an indirect relationship caused by a hidden variable. For example, a recent study in flowering plants suggests that environmental energy has strong independent effects on both substitution rate and speciation rate [64].

## Conclusion

We have shown that deep metazoan lineages differ systematically in rate of molecular evolution for mitochondrial genes. Importantly, our results suggest that if body size, or possibly net diversification rate, have shown consistent trends during the radiation of the Metazoa, then it is possible that many lineages could have undergone concerted changes in rate of molecular evolution. Such a concerted change could potentially confound attempts to date the metazoan radiation using mitochondrial protein-coding genes, whether by traditional molecular clock analyses or the more recent rate-variable methods.

Our results also suggest that molecular dates might be overestimated when larger animals are over-represented in the sampled taxa. Such a bias could apply even if the body size of Metazoa has not systematically increased. It is also important to note that our results apply only to mitochondrial genes, yet Precambrian molecular dates have predominantly been generated from nuclear gene data.

However, our results do suggest that the search for biological correlates of molecular rates may uncover important patterns that may be used to assess the reliability of molecular dates, or develop new dating methods that can incorporate prior knowledge of molecular evolutionary rates. In particular, this study emphasizes the importance of examining patterns of molecular evolution at different "depths" in a phylogeny, as a pattern evident in deep comparisons may not be detectable for shallow pairs.

## Methods

### Comparison of phylogenetically independent sister taxa

To explore the relationship between lineage-specific rates of molecular evolution and body size or net diversification rate, we used the method of phylogenetically independent comparisons [65,66]. Such methods are necessary due to the common ancestry of taxa, which implies that measurements from different taxa are not statistically independent. Ignoring this fact means that the same evolutionary history may be counted multiple times, and this could generate a spurious association between variables [66]. As such, we take as each datapoint the proportional change in the chosen variables between a pair of sister taxa. Each pair is chosen so as not to overlap on the phylogeny with any other pair, ensuring that the datapoints are statistically independent [37,38].

### Choice of taxa

We collected data for 64 major taxa in the metazoan phylogeny (Table 2). Phylogenetically independent sister pairs were chosen from these 64 using an assumed phylogeny (see Figure 1), constructed from multiple sources of evidence from the literature (in particular from [24,44-47]. Because aspects of metazoan phylogeny remain controversial, we avoided including taxa whose placement varied between our sources. If aspects of this phylogeny are found to be incorrect in future, we may have to revise some data points, but we are confident that on the whole we have been able to choose phylogenetically independent comparisons (Table 2). Because we have restricted the analysis to sister pairs, divergence dates for the pairs were not required. Deep phylogenetic uncertainties, such as the position of the Mollusca, did not affect the choice of the pairs. The comparison depth was chosen to optimize DNA sequence availability and number of comparisons, targeting lineages relevant to dating the Cambrian explosion.

**Table 2:** Body size and species number values from Orme et al. [43] for each comparison pair. Comparisons 1 to 25 are the default set used for most genes (see Figure 1). Comparisons 26 to 29 are additional pairs used when sequence was unavailable for one or more of the default set.

	**Taxon 1**	**Taxon 2**	**Biovolume 1 (mm^3^)**	**Biovolume 2 (mm^3^)**	**Species number 1**	**Species number 2**
**1**	Acoela	Orthonectida	2.10E-02	1.30E-04	319	20
**2**	Turbellaria	Trematoda	1.32E+01	1.25E-01	15000	20000
**3**	Cestoda	Monogenea	1.12E+01	8.20E-02	10000	10000
**4**	Urochordata	Cephalochordata	4.50E+03	2.31E+02	1990	25
**5**	Chondrichthyes	Petromyzontidae	5.30E+07	9.84E+05	848	84
**6**	Serpentes	Lepidosauria	1.13E+05	5.50E+03	2500	3000
**7**	Echinoidea	Holothuroidea	1.93E+05	1.64E+04	950	1150
**8**	Asteroidea	Ophiuroidea	1.72E+05	6.28E+03	1500	2000
**9**	Enteropneusta	Pterobranchia	1.56E+03	6.60E+00	70	25
**10**	Priapulida	Kinorhyncha	6.35E+03	1.10E-03	17	150
**11**	Nematomorpha	Nematoda	8.33E+01	2.50E-03	304	20000
**12**	Onychophora	Tardigrada	8.51E+02	2.60E-03	70	600
**13**	Araneae	Acari	8.82E+01	2.01E+01	37000	45000
**14**	Orthoptera	Hemiptera	4.80E+02	4.25E+01	20000	98000
**15**	Hymenoptera	Coleoptera	5.00E+01	4.43E+01	120000	350000
**16**	Lepidoptera	Diptera	1.62E+02	9.40E+00	160000	120000
**17**	Copepoda	Ostracoda	6.90E-02	1.60E-02	9000	8000
**18**	Eucarida	Peracarida	1.69E+04	3.20E+01	10566	12706
**19**	Brachiopoda	Phoroniformea	5.18E+03	5.80E+02	335	12
**20**	Acanthocephala	Rotifera	8.40E+00	3.20E-03	900	1800
**21**	Aplacophora	Caudofoveata	1.56E+02	5.47E+01	180	70
**22**	Bivalvia	Polyplacophora	2.72E+03	8.58E+02	20000	550
**23**	Cephalopoda	Scaphopoda	7.33E+04	4.86E+00	656	350
**24**	Opisthobranchia	Pulmonata	2.21E+02	9.03E+01	1000	20000
**25**	Polychaeta	Oligochaeta	4.80E+02	1.86E+01	12000	6000

**26**	Petromyzontidae	Urochordata	9.84E+05	4.50E+03	84	1990
**27**	Echinoidea	Asteroidea	1.93E+05	1.72E+05	950	1500
**28**	Priapulida	Nematoda	6.35E+03	2.50E-03	17	20000
**29**	Cestoda	Trematoda	1.12E+01	1.25E-01	10000	20000

### Body size and net diversification rate data

To represent the variables of body size and net diversification rate, we used estimates of median adult biovolume and extant species number presented in Orme *et al*. ([43]: Table 1). In their study, biovolume (in mm^3^) was calculated for each taxon as the product of three linear dimensions – length, width and height – obtained from the literature. For each linear dimension, the authors use the median as a measure of central tendency, because of the left skewed distribution of most of the taxa. For example, for the Nemertea, the median length is 75 mm but the arithmetic mean is 323 mm, largely because of the bootlace worm (*Lineus longissimus*) which can be up to 10 metres long. When one or two of these linear dimensions were not available, Orme *et al*. extrapolated missing values from the available dimensions and the body form. Net diversification rate (the net result of speciation and extinction for each taxon) is represented by the estimated number of extant species described per taxon (here referred to as "clade size"). When several estimates were available and differed between sources, Orme *et al*. chose the most recent estimates, or the estimates from studies which focus on the particular taxa.

### Substitution rate data

To estimate change in rate of molecular evolution for each of our species pairs, DNA sequence data from one species from each taxon was collected from GenBank ([67]; for details of sequences and taxons, see Additional files 5 and 6). When several equally complete sequences were available for a taxon, one was chosen at random. Because sequences of different types may show different patterns of evolution, separate results are reported for six mitochondrial protein-coding genes (COI, COII, COIII, CytB, NADH1, NADH2), a nuclear protein-coding gene (ef1a) and two nuclear RNA-coding genes (18S, 28S). However, not all genes were available for all taxa [for details of sequences, species names and accessions numbers, see Additional file 7].

The DNA sequences were automatically aligned using ClustalW [68]. These alignments were manually refined using BioEdit [69] and the unalignable parts excised: between 11% and 70% of the original sequences were removed depending on the gene [alignments are available from the authors and for details of the excised part, see Additional file 8]. In addition to analysing each gene separately, a concatenated alignment of the six mitochondrial genes was generated, and if any taxon was missing a gene it was replaced by gaps. Because some concatenated sequences included a relatively high proportion of gaps that might bias branch length estimation, in addition to the complete concatenated alignment (C100), we generated a further alignment (C20) where pairs were removed if they contained over 20% of missing sequence. Four pairs were removed under this criterion [see Additional file 9].

Fixing the phylogeny to the assumed relationships (see Figure 1), we estimated branch lengths by maximum likelihood, using the BASEML software from PAML v3.15 [70]. For each sequence, we chose the HKY+Γ model of DNA substitution, with parameter values estimated from the data for each alignment. Because our phylogeny contained long terminal branches and shorter internal branches, the use of a more parameter-rich model might lead to overparametrization, which can bias branch length estimates [71,72]. For the protein-coding sequences, codon-based models could not be used, as our phylogeny contains multiple genetic codes. However, for these genes, separate results are reported for branch lengths estimated under a partition of the data into the three codon positions. For the concatenated alignment of six mitochondrial genes, results are also reported for a partition in which each gene was assigned its own rate. The computed values of the branch length estimates in each pair of sister taxa are available in Additional files 5 and 6. To estimate the proportional change in the rate of molecular evolution between a pair of sister taxa, we used the ratio of their maximum likelihood branch lengths. Each branch length is the product of a rate of evolution and a divergence time, so for pairs of sister taxa which share the same divergence time, time cancels from the estimate with the use of the ratio.

### Statistical tests

Because we cannot assume any particular distribution for the error associated with the variables under study (molecular rate, body size and clade size), we used the non-parametric Spearman's rank correlation test to explore their association. This test takes into account the magnitude of the difference in each variable, but makes no detailed assumptions about their associated error. The tests were two-tailed, as we did not wish to assume the direction of any potential association between rate and traits. Tests were conducted using the programming language R [73], with PAML output initially formatted using the phylogenetic package Ape [74] available for R.

The Spearman's rank correlation test was used to test for associations between the relative substitution rate variable and the biological traits variable (biovolume or clade size). For each pair of taxa, the biological traits variable is the ratio of the bigger over the smaller biological trait value. The relative substitution rate variable was represented by the ratio *BL*_Big _*/BL*_Small _where for a pair of taxa, *BL*_Big _and *BL*_Small _represent the branch lengths of the taxon with, respectively, the larger and smaller values of the relevant trait (biovolume or species number).

## Authors' contributions

LB designed research project. EF and JT collected data, EF and JJW performed analyses. All authors wrote and approved the final MS.

## Supplementary Material

Additional file 1 Taxonomic ranks of the taxa under study. Click here for file

Additional file 2 **Scatterplots of the log of the relative molecular rate variable (y axis) versus the log of the relative biovolume variable (x axis) for each comparison pair. **BB and SB represent respectively the bigger and smaller biovolumes; BL_BB and BL_SB represent the branch lengths of the taxon with, respectively, the larger and smaller biovolumes. Dotted lines indicate the median values of the variables. The branch lengths were estimated using three models: NP: no rate partition in the data; CP: codon position partitions for the protein-coding genes; GP: gene partitions for the concatenation of mitochondrial genes. (a): nuclear genes; (b): mitochondrial genes; (c): concatenations of mitochondrial genes. Click here for file

Additional file 3 **Scatterplots of the log of the relative molecular rate variable (y axis) versus the log of the species number variable (x axis) for each comparison pair. **BN and SN represent respectively the bigger and smaller species number; BL_BN and BL_SN represent the branch lengths of the taxon with, respectively, the larger and smaller species number. Dotted lines indicate the median values of the variables. The branch lengths were estimated using three models: NP: no rate partition in the data; CP: codon position partitions for the protein-coding genes; GP: gene partitions for the concatenation of mitochondrial genes. (a): nuclear genes; (b): mitochondrial genes; (c): concatenations of mitochondrial genes. Click here for file

Additional file 4 **Comparison of body size ratios used in the present study with those used by Thomas *et al*.**[38]. Shown for several taxa are the minimum ratio, the maximum ratio and geometric mean ratio. Click here for file

Additional file 5 **Branch length estimates and body size values (in mm**^3^**). **Body size values are taken from Orme *et al*. [43]. Click here for file

Additional file 6 **Branch length estimates and species richness values. **Species richness values are taken from Orme *et al*. [43]. Click here for file

Additional file 7 **Pairs used for each gene under analysis. **Each sequence that represents a taxon is reported together with its accession number from GenBank with the species it came from. The 25 first pairs represent the original pairs chosen on the metazoan tree (see Figure 1). Because gene sequences are not available for each pair, we chose additional phylogenetically independent pairs for certain genes (pairs 26 to 29). The symbol "-" indicates that gene sequences were not available for either one or both members of a comparison pair. Click here for file

Additional file 8 **Alignment length before and after removing unalignable sequence. **Bases were excised manually in Bioedit [69]. Click here for file

Additional file 9 **Comparison pairs used for each gene. **Each pair used is indicated by an 'X'. Also shown are the total number of comparison pairs used, and the alignment lengths in base pairs. Click here for file
