# Supplementary material for: The influence of body size and net diversification rate on molecular evolution during the radiation of animal phyla
Source: BMC Evol Biol. 2007 Jun 26;7:95. doi: 10.1186/1471-2148-7-95 (PMC1929056; doi:10.1186/1471-2148-7-95)
Supplement: Additional file 1 — Taxonomic ranks of the taxa under study. [file 1471-2148-7-95-S1.pdf]

Acoela	**Order**	Orthonectida	**Phylum**
Turbellaria	**Class**	Trematoda	**Class**
Cestoda	**Class**	Monogenea	**Class**
Urochordata	**Sub-phylum**	Cephalochordata	**Sub-phylum**
Chondrichthyes	**Class**	Petromyzontidae	**Family**
Serpentes	**Infra-order**	Lepidosauria	**Super-order**
Echinoidea	**Class**	Holothuroidea	**Class**
Asteroidea	**Class**	Ophiuroidea	**Class**
Enteropneusta	**Class**	Pterobranchia	**Class**
Priapulida	**Phylum**	Kinorhyncha	**Phylum**
Nematomorpha	**Phylum**	Nematoda	**Phylum**
Onychophora	**Phylum**	Tardigrada	**Phylum**
Araneae	**Order**	Acari	**Sub-class**
Orthoptera	**Order**	Hemiptera	**Order**
Hymenoptera	**Order**	Coleoptera	**Order**
Lepidoptera	**Order**	Diptera	**Order**
Copepoda	**Sub-class**	Ostracoda	**Class**
Eucarida	**Super-order**	Peracarida	**Super-order**
Brachiopoda	**Phylum**	Phoroniformea	**Sub-phylum**
Acanthocephala	**Phylum**	Rotifera	**Phylum**
Aplacophora	**Class**	Caudofoveata	**Sub-class**
Bivalvia	**Class**	Polyplacophora	**Class**
Cephalopoda	**Class**	Scaphopoda	**Class**
Opisthobranchia	**Order**	Pulmonata	**Order**
Polychaeta	**Class**	Oligochaeta	**Sub-class**
