# Supplementary figures and images for: The influence of body size and net diversification rate on molecular evolution during the radiation of animal phyla
Source: BMC Evol Biol. 2007 Jun 26;7:95. doi: 10.1186/1471-2148-7-95 (PMC1929056; doi:10.1186/1471-2148-7-95)

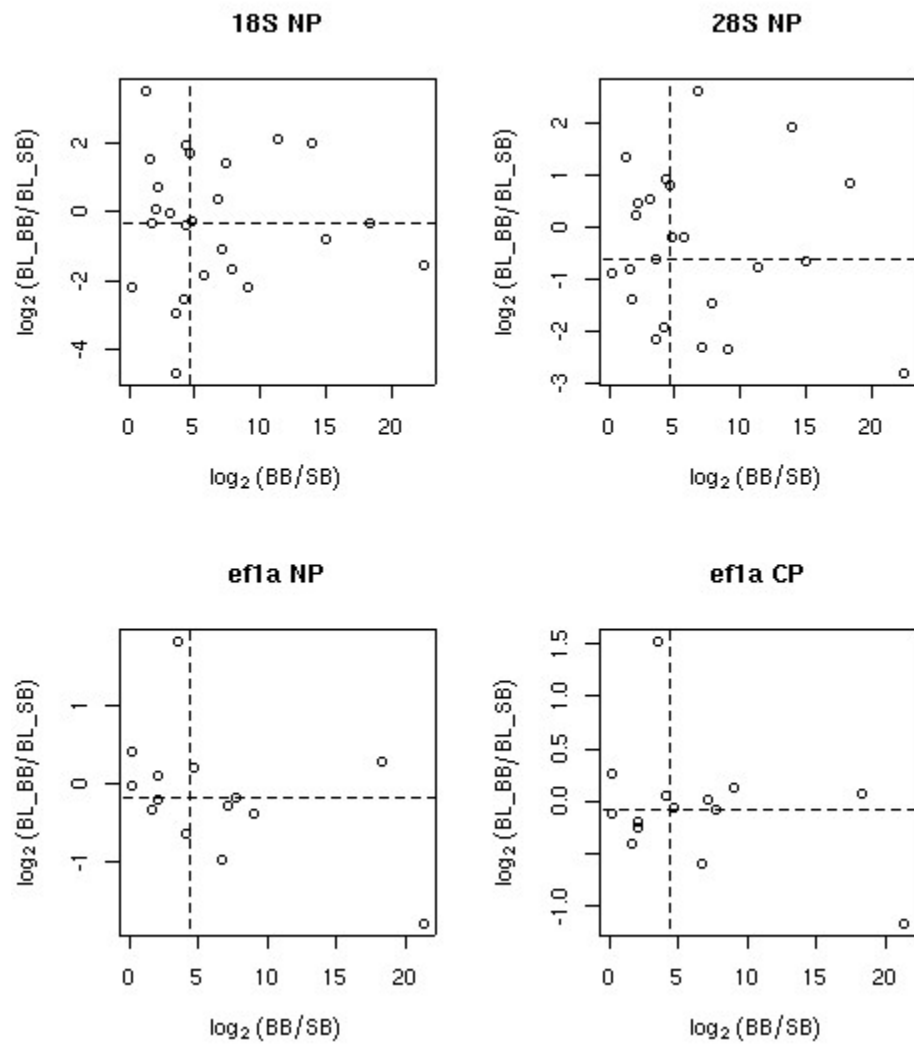

**(a) Nuclear genes**

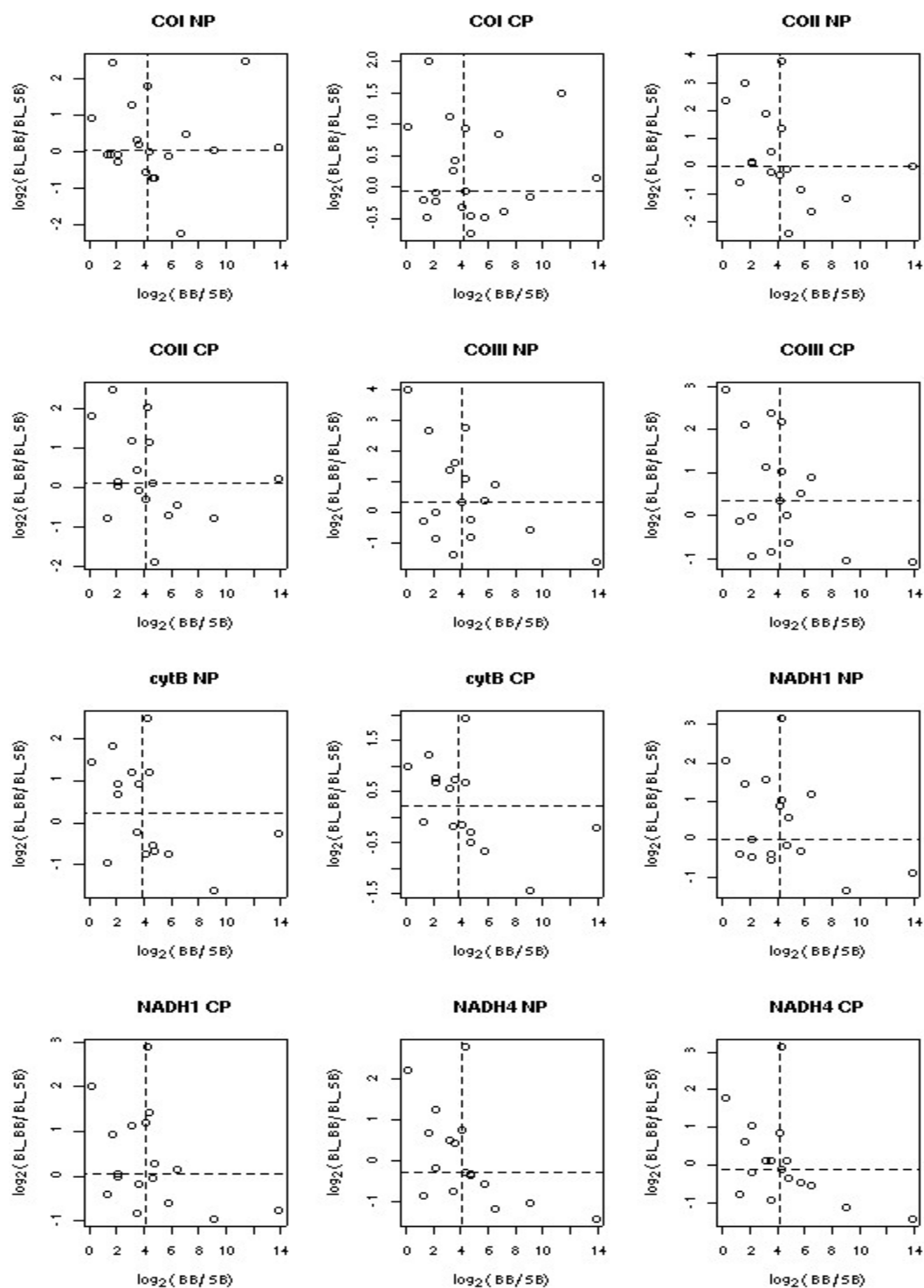

(b) Mitochondrial genes

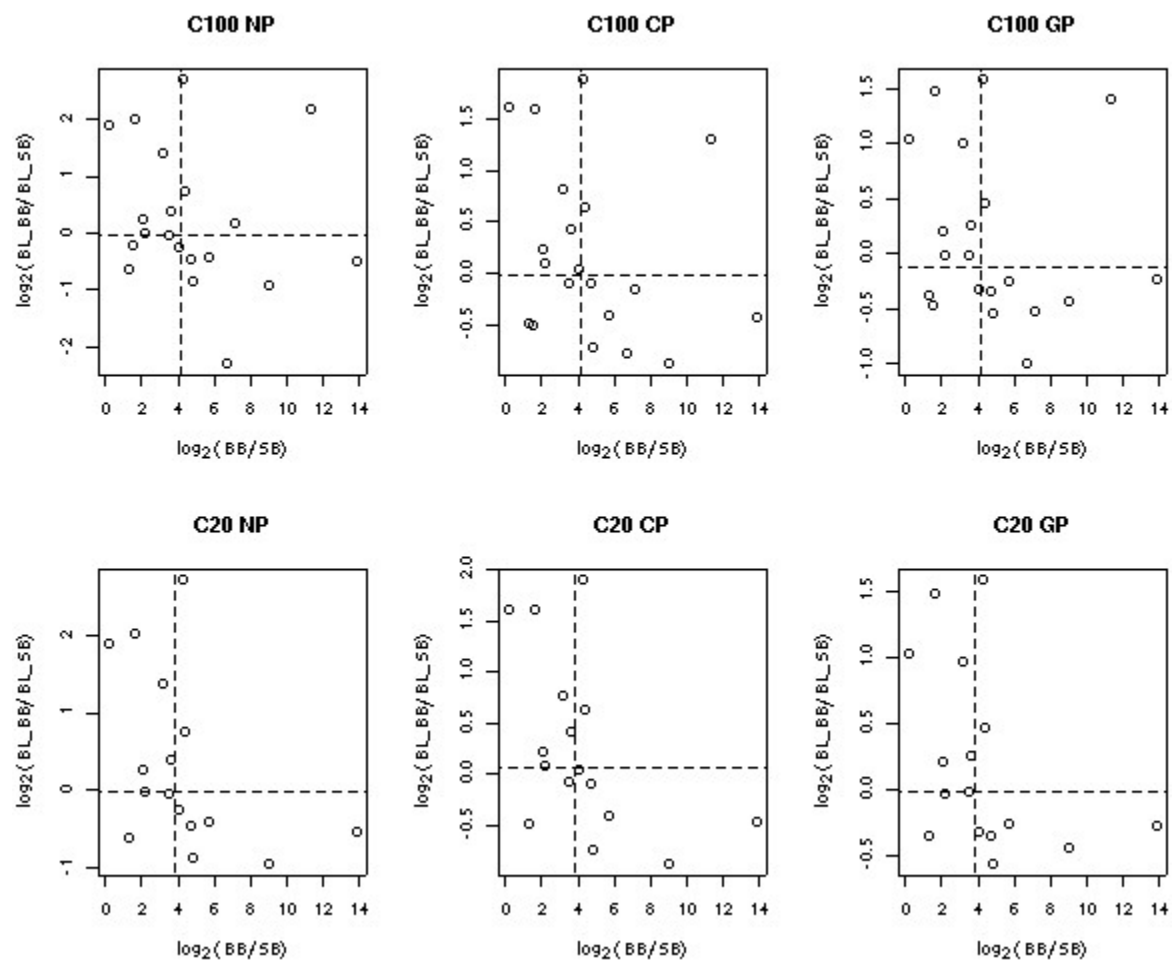

**(c) Concatenation of the mitochondrial genes**

Supplement: Additional file 2 — Scatterplots of the log of the relative molecular rate variable (y axis) versus the log of the relative biovolume variable (x axis) for each comparison pair. BB and SB represent respectively the bigger and smaller biovolumes; BL_BB and BL_SB represent the branch lengths of the taxon with, respectively, the larger and smaller biovolumes. Dotted lines indicate the median values of the variables. The branch lengths were estimated using three models: NP: no rate partition in the data; CP: codon position partitions for the protein-coding genes; GP: gene partitions for the concatenation of mitochondrial genes. (a): nuclear genes; (b): mitochondrial genes; (c): concatenations of mitochondrial genes. [file 1471-2148-7-95-S2.pdf]

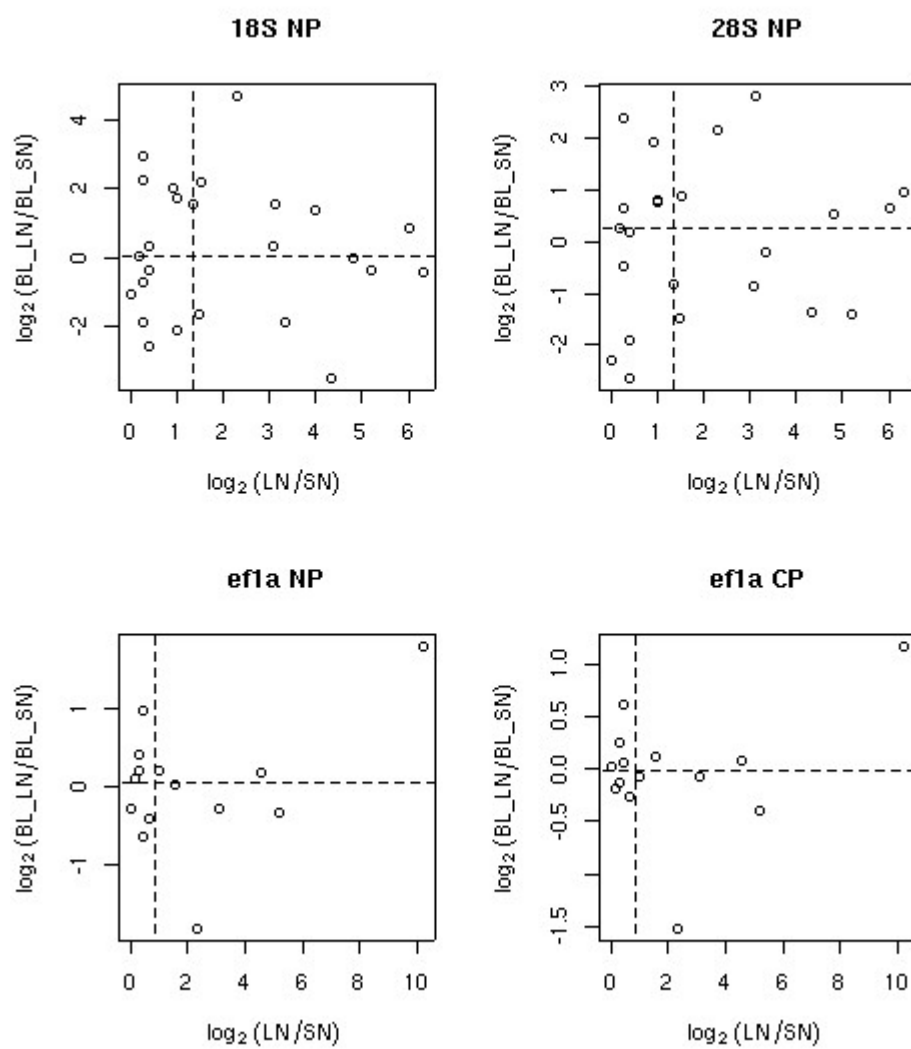

**(a) Nuclear genes**

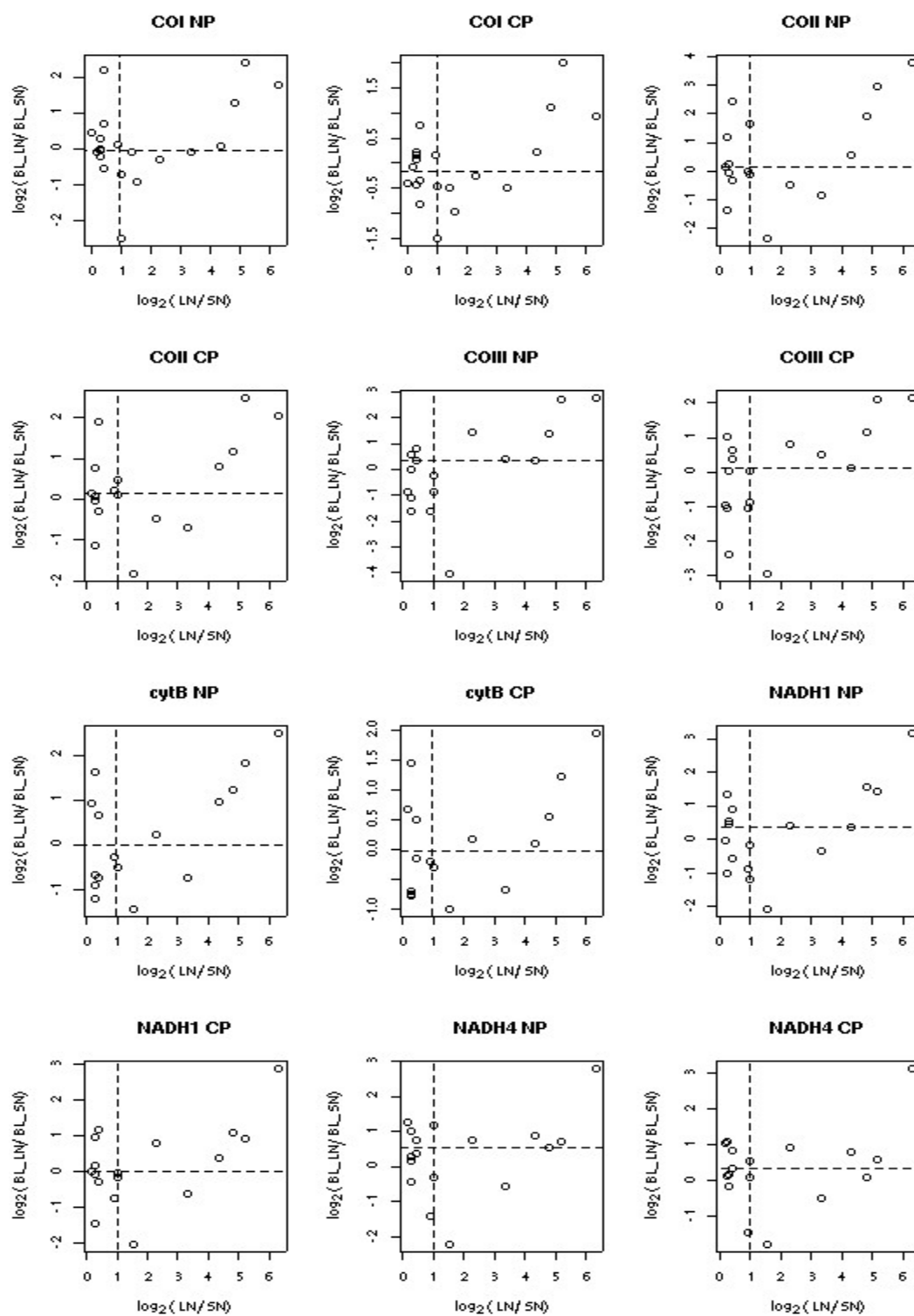

(b) Mitochondrial genes

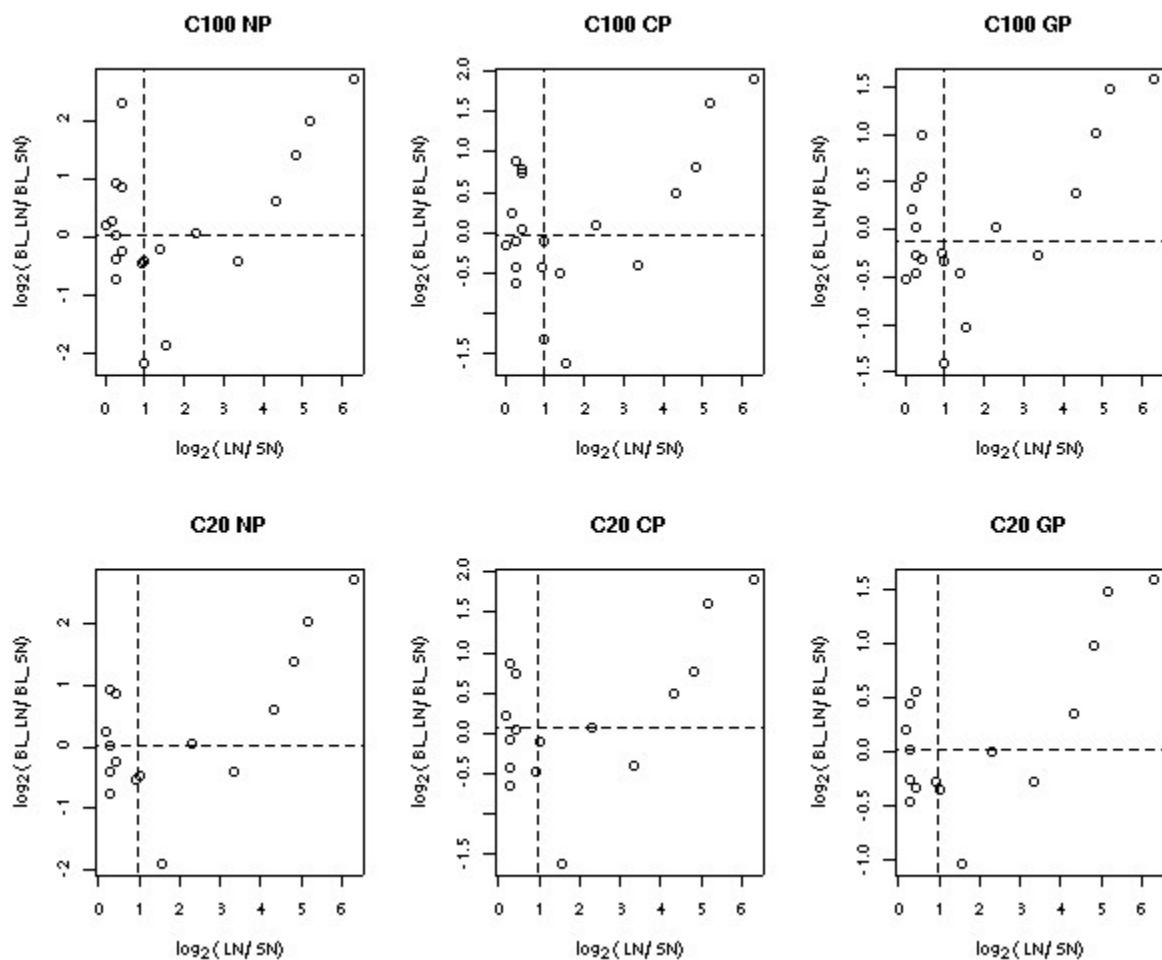

(c) Concatenation of the mitochondrial genes

Supplement: Additional file 3 — Scatterplots of the log of the relative molecular rate variable (y axis) versus the log of the species number variable (x axis) for each comparison pair. BN and SN represent respectively the bigger and smaller species number; BL_BN and BL_SN represent the branch lengths of the taxon with, respectively, the larger and smaller species number. Dotted lines indicate the median values of the variables. The branch lengths were estimated using three models: NP: no rate partition in the data; CP: codon position partitions for the protein-coding genes; GP: gene partitions for the concatenation of mitochondrial genes. (a): nuclear genes; (b): mitochondrial genes; (c): concatenations of mitochondrial genes. [file 1471-2148-7-95-S3.pdf]
