# Supplementary material for: The influence of body size and net diversification rate on molecular evolution during the radiation of animal phyla
Source: BMC Evol Biol. 2007 Jun 26;7:95. doi: 10.1186/1471-2148-7-95 (PMC1929056; doi:10.1186/1471-2148-7-95)
Supplement: Additional file 4 — Comparison of body size ratios used in the present study with those used by Thomas et al.[38]. Shown for several taxa are the minimum ratio, the maximum ratio and geometric mean ratio. [file 1471-2148-7-95-S4.pdf]

		Min	Max	Geometric Mean
**Thomas *et al.* (1)**	**Lepidoptera**	1.00	2.70	1.26
	**Arachnida**	1.17	5.05	1.74
	**Cephalopoda**	1.10	150.00	2.89
	**Gastropoda**	1.31	25.05	3.78
	**Echinodermata**	1.10	7.00	2.06
	**Platyhelminthes**	1.03	5000.00	7.71
	**Hymenoptera**	1.10	17.71	3.27
	**Annelida**	1.02	8.38	3.20
	**Bivalvia**	1.26	49.79	5.44
	**Monogenea**	1.18	9.11	2.09
**Present study**	**Metazoa**	1.13	5775454.55	124.67
