# Supplementary material for: The influence of body size and net diversification rate on molecular evolution during the radiation of animal phyla
Source: BMC Evol Biol. 2007 Jun 26;7:95. doi: 10.1186/1471-2148-7-95 (PMC1929056; doi:10.1186/1471-2148-7-95)
Supplement: Additional file 7 — Pairs used for each gene under analysis. Each sequence that represents a taxon is reported together with its accession number from GenBank with the species it came from. The 25 first pairs represent the original pairs chosen on the metazoan tree (see Figure 1). Because gene sequences are not available for each pair, we chose additional phylogenetically independent pairs for certain genes (pairs 26 to 29). The symbol "-" indicates that gene sequences were not available for either one or both members of a comparison pair. [file 1471-2148-7-95-S7.pdf]

Quick access to the tables:

18S, 28S, ef1a, COI, COII, COIII, cytB, NADH1, NADH4

(a) : 18S

	Pairs (Taxon 1/Taxon 2)	Taxon 1 Species name	Taxon 1 Acc. No.	Taxon 2 Species name	Taxon 2 Acc. No.
1	Acoela/Orthonectida	*Symsagittifera corsicae*	AJ319029	*Rhopalura ophiocomae*	X97158
2	Turbellaria/Trematoda	*Archimonocelis crucifera*	AJ270151	*Xystretum sp.*	AJ287588
3	Cestoda/Monogenea	*Skrjabinia cesticillus*	AY382316	*Haliotrema scyphovagina*	AY820611
4	Urochordata/Cephalochordata	*Megalodicopia hians*	AB075543	*Branchiostoma floridae*	M97571
5	Chondrichthyes/Petromyzontidae	*Urobatis jamaicensis*	AY049861	*Petromyzon marinus*	M97575
6	Serpentes/Lepidosauria	*Heterodon platirhinos*	M59392	*Sceloporus undulatus*	M59400
7	Echinoidea/Holothuroidea	*Temnopleurus hardwickii*	Z37135	*Afrocucumis africana*	AY133483
8	Asteroidea/Ophiuroidea	*Asterina coronata*	AB084566	*Ophiocanops fugiens*	Z80954
9	Enteropneusta/Pterobranchia	*Harrimania planktophilus*	AF236799	*Cephalodiscus gracilis*	AF236798
10	Priapulida/Kinorhyncha	*Tubiluchus corallicola*	AF119086	*Pycnophyes kielensis*	U67997
11	Nematomorpha/Nematoda	*Gordius sp.*	U51005	*Stilbonema majum*	Y16922
12	Onychophora/Tardigrada	*Euperipatoides leuckartii*	U49910	*Hypsibius sp.*	Z93337
13	Araneae/Acari	*Nesticus cellulanus*	AF005447	*Opilioacarus texanus*	AF115375
14	Orthoptera/Hemiptera	*Oxya chinensis*	AY037173	*Henestaris oschanini*	AY324853
15	Hymenoptera/Coleoptera	*Aphelinus gossypii*	AY216700	*Clambus arnetti*	AF012526
16	Lepidoptera/Diptera	*Attacus ricini*	AF535029	*Anopheles maculatus*	AF440198
17	Copepoda/Ostracoda	*Lepeophtheirus salmonis*	AF208263	*Heterocypris sp.*	L81944
18	Eucarida/Peracarida	*Jasus edwardsii*	AF235972	*Storthyngurella triplospinosa*	AY461482
19	Brachiopoda/Phoroniformea	*Argyrotheca cordata*	AF119078	*Phoronis australis*	AF119079
20	Acanthocephala/Rotifera	*Polyacanthorhynchus caballeroi*	AF388660	*Brachionus patulus*	AF154568
21	Aplacophora/Caudofoveata	*Helicoradomenia sp.*	AY212108	*Scutopus ventrolineatus*	X91977
22	Bivalvia/Polyplacophora	*Bathypecten vulcani*	AY557608	*Acanthochitona crinita*	AF120503
23	Cephalopoda/Scaphopoda	*Psychroteuthis sp.*	AY557513	*Rhabdus rectius*	AF120523
24	Opisthobranchia/Pulmonata	*Notaeolidia depressa*	AY165770	*Lymnaea viatrix*	AY057088
25	Polychaeta/Oligochaeta	*Proceraea cornuta*	AF212179	*Stylaria sp.*	U95946
26	Petromyzontidae/Urochordata	-	-	-	-
27	Echinoidea/Asteroidea	-	-	-	-
28	Priapulida/Nematoda	-	-	-	-
29	Cestoda/Trematoda	-	-	-	-

28S (next)   Legend

(b) : 28S

Pairs (Taxon 1/Taxon 2)		Taxon 1 Species name	Taxon 1 Acc. No.	Taxon 2 Species name	Taxon 2 Acc. No.
1	Acoela/Orthonectida	-	-	-	-
2	Turbellaria/Trematoda	*Childia groenlandica*	AY157603	*Lobatostoma manteri*	AY157177
3	Cestoda/Monogenea	*Raillietina australis*	AF286914	*Gyrodactylus salaris*	AJ542394
4	Urochordata/Cephalochordata	*Ciona intestinalis*	AF212177	*Branchiostoma floridae*	AF061796
5	Chondrichthyes/Petromyzontidae	*Rhinobatos productus*	AY049851	*Petromyzon marinus*	AF061798
6	Serpentes/Lepidosauria	-	-	-	-
7	Echinoidea/Holothuroidea	*Arbacia punctulata*	AY026367	*Psychropotes longicauda*	Z80946
8	Asteroidea/Ophiuroidea	*Porania pulvillus*	Z80945	*Ophiocanops fugiens*	Z80943
9	Enteropneusta/Pterobranchia	*Saccoglossus kowalevskii*	AF212175	*Cephalodiscus gracilis*	AF212172
10	Priapulida/Kinorhyncha	*Priapulus caudatus*	AY210840	*Pycnophyes greenlandicus*	AY428828
11	Nematomorpha/Nematoda	*Gordius aquaticus*	AY210817	*Labiostrongylus bipapillosus*	AJ512837
12	Onychophora/Tardigrada	*Peripatus sp.*	AY210836	*Milnesium sp.*	AY210826
13	Araneae/Acari	*Misumenops asperatus*	AY210461	*Boophilus microplus*	AF200189
14	Orthoptera/Hemiptera	*Melanoplus sp.*	AY125286	*Euritea munda*	AF304633
15	Hymenoptera/Coleoptera	*Ussurohelcon nigricornis*	AJ302912	*Toramus sp.*	AY310676
16	Lepidoptera/Diptera	*Hemileuca sp.*	AF423922	*Simulium sanctipauli*	AF403820
17	Copepoda/Ostracoda	*Cyclopidae sp.*	AY210813	*Cyprididae sp.*	AY210815
18	Eucarida/Peracarida	*Aegla humahuaca*	AY596086	*Porcellio scaber*	AY744901
19	Brachiopoda/Phoroniformea	*Glottidia pyramidata*	AY210459	*Phoronis vancouverensis*	AF342797
20	Acanthocephala/Rotifera	*Oligacanthorhynchus tortuosa*	AY210466	*Philodina roseola*	AY210469
21	Aplacophora/Caudofoveata	*Helicoradomenia sp.*	AY145409	*Chaetoderma sp.*	AY145397
22	Bivalvia/Polyplacophora	*Solemya velum*	AY145421	*Chaetopleura apiculata*	AY145398
23	Cephalopoda/Scaphopoda	*Histioteuthis sp.*	AY145410	*Dentalium octangulatum*	AY145403
24	Opisthobranchia/Pulmonata	*Aplysia californica*	AY026366	*Deroceras reticulatum*	AY145404
25	Polychaeta/Oligochaeta	*Nereis succinea*	AY210464	*Eisenia fetida*	AF212166
26	Petromyzontidae/Urochordata	-	-	-	-
27	Echinoidea/Asteroidea	-	-	-	-
28	Priapulida/Nematoda	-	-	-	-
29	Cestoda/Trematoda	-	-	-	-

18S (previous) ef1a (next) Legend

(c) : ef1a

	Pairs (Taxon 1/Taxon 2)	Taxon 1		Taxon 2	
		Species name	Acc. No.	Species name	Acc. No.
1	Acoela/Orthonectida	-	-	-	-
2	Turbellaria/Trematoda	*Mesostoma lingua*	AF288069	*Schistosoma japonicum*	AF288067
3	Cestoda/Monogenea	*Grillotia erinaceus*	AF288066	*Neomicrocotyle pacifica*	AF288070
4	Urochordata/Cephalochordata	-	-	-	-
5	Chondrichthyes/Petromyzontidae	-	-	-	-
6	Serpentes/Lepidosauria	-	-	-	-
7	Echinoidea/Holothuroidea	-	-	-	-
8	Asteroidea/Ophiuroidea	-	-	-	-
9	Enteropneusta/Pterobranchia	-	-	-	-
10	Priapulida/Kinorhyncha	-	-	-	-
11	Nematomorpha/Nematoda	-	-	-	-
12	Onychophora/Tardigrada	*Euperipatoides rowelli*	AF137394	*Milnesium tardigradum*	AF063419
13	Araneae/Acari	*Aphonopelma chalcodes*	U90045	*Calyptophthiracarus olivaceus*	AF240853
14	Orthoptera/Hemiptera	*Locusta migratoria*	AY077627	*Stictopelta sp.*	AF182579
15	Hymenoptera/Coleoptera	*Leioproctus sp.*	AY585141	*Micracis carinulatus*	AF186677
16	Lepidoptera/Diptera	*Saturnia pyri*	DQ077816	*Ectemnia sp.*	AF003589
17	Copepoda/Ostracoda	*Eurytemora affinis*	AF063408	*Cypridopsis vidua*	AF063414
18	Eucarida/Peracarida	*Libinia emarginata*	U90050	*Heteromysis formosa*	AF063410
19	Brachiopoda/Phoroniformea	-	-	-	-
20	Acanthocephala/Rotifera	-	-	-	-
21	Aplacophora/Caudofoveata	-	-	-	-
22	Bivalvia/Polyplacophora	*Mytilus galloprovincialis*	AB162021	*Chaetopleura apiculata*	U90062
23	Cephalopoda/Scaphopoda	-	-	-	-
24	Opisthobranchia/Pulmonata	-	-	-	-
25	Polychaeta/Oligochaeta	*Chaetopterus sp.*	AY580184	*Enchytraeus sp.*	AF063418
26	Petromyzontidae/Urochordata	*Oikopleura dioica*	AF468038	*Lethenteron japonicum*	AB183717
27	Echinoidea/Asteroidea	*Strongylocentrotus purpuratus*	AY580285	*Asterina miniata*	AY580177
28	Priapulida/Nematoda	*Priapulus caudatus*	AY580299	*Onchocerca volvulus*	M64333
29	Cestoda/Trematoda	-	-	-	-

28S (previous) COI (next) Legend

(d) : COI

Pairs (Taxon 1/Taxon 2)		Taxon 1 Species name	Taxon 1 Acc. No.	Taxon 2 Species name	Taxon 2 Acc. No.
1	Acoela/Orthonectida	-	-	-	-
2	Turbellaria/Trematoda	*Pseudostylochus intermedius*	AB049114	*Paragonimus westermani*	NC_002354
3	Cestoda/Monogenea	*Taenia asiatica*	AF445798	*Gyrodactylus salaris*	AF479750
4	Urochordata/Cephalochordata	*Doliolum nationalis*	NC_006627	*Epigonichthys lucayanus*	AB110092
5	Chondrichthyes/Petromyzontidae	*Scyliorhinus canicula*	NC_001950	*Petromyzon marinus*	NC_001626
6	Serpentes/Lepidosauria	*Leptotyphlops dulcis*	NC_005961	*Teratoscincus keyserlingii*	NC_007008
7	Echinoidea/Holothuroidea	*Arbacia lixula*	NC_001770	*Cucumaria miniata*	NC_005929
8	Asteroidea/Ophiuroidea	*Pisaster ochraceus*	NC_004610	*Ophiura lutkeni*	NC_005930
9	Enteropneusta/Pterobranchia	-	-	-	-
10	Priapulida/Kinorhyncha	-	-	-	-
11	Nematomorpha/Nematoda	-	-	-	-
12	Onychophora/Tardigrada	-	-	-	-
13	Araneae/Acari	*Heptathela hangzhouensis*	NC_005924	*Ixodes uriae*	NC_006078
14	Orthoptera/Hemiptera	*Locusta migratoria*	NC_001712	*Philaenus spumarius*	NC_005944
15	Hymenoptera/Coleoptera	*Melipona bicolor*	NC_004529	*Crioceris duodecimpunctata*	NC_003372
16	Lepidoptera/Diptera	*Antheraea pernyi*	NC_004622	*Bactrocera oleae*	NC_005333
17	Copepoda/Ostracoda	*Tigriopus japonicus*	NC_003979	*Vargula hilgendorfii*	NC_005306
18	Eucarida/Peracarida	*Marsupenaeus japonicus*	NC_007010	*Parhyale hawaiiensis*	AY639937
19	Brachiopoda/Phoroniformea	*Terebratalia transversa*	NC_003086	*Phoronis psammophila*	AY368231
20	Acanthocephala/Rotifera	*Leptorhynchoides thecatus*	NC_006892	*Synchaeta cf. pectinata*	AF499093
21	Aplacophora/Caudofoveata	*Helicoradomenia sp.*	AY377725	*Chaetoderma nitidulum*	AY377726
22	Bivalvia/Polyplacophora	*Mytilus galloprovincialis*	NC_006886	*Katharina tunicata*	NC_001636
23	Cephalopoda/Scaphopoda	*Todarodes pacificus*	NC_006354	*Graptacme eborea*	NC_006162
24	Opisthobranchia/Pulmonata	*Aplysia californica*	NC_005827	*Albinaria caerulea*	NC_001761
25	Polychaeta/Oligochaeta	*Clymenella torquata*	NC_006321	*Lumbricus terrestris*	NC_001673
26	Petromyzontidae/Urochordata	-	-	-	-
27	Echinoidea/Asteroidea	-	-	-	-
28	Priapulida/Nematoda	-	-	-	-
29	Cestoda/Trematoda	-	-	-	-

ef1a (previous) COII (next) Legend

(e) : COII

	Pairs (Taxon 1/Taxon 2)	Taxon 1		Taxon 2	
		Species name	Acc. No.	Species name	Acc. No.
1	Acoela/Orthonectida	-	-	-	-
2	Turbellaria/Trematoda	-	-	-	-
3	Cestoda/Monogenea	-	-	-	-
4	Urochordata/Cephalochordata	*Doliolum nationalis*	NC_006627	*Epigonichthys lucayanus*	AB110092
5	Chondrichthyes/Petromyzontidae	*Scyliorhinus canicula*	NC_001950	*Petromyzon marinus*	NC_001626
6	Serpentes/Lepidosauria	*Leptotyphlops dulcis*	NC_005961	*Teratoscincus keyserlingii*	NC_007008
7	Echinoidea/Holothuroidea	*Arbacia lixula*	NC_001770	*Cucumaria miniata*	NC_005929
8	Asteroidea/Ophiuroidea	*Pisaster ochraceus*	NC_004610	*Ophiura lutkeni*	NC_005930
9	Enteropneusta/Pterobranchia	-	-	-	-
10	Priapulida/Kinorhyncha	-	-	-	-
11	Nematomorpha/Nematoda	-	-	-	-
12	Onychophora/Tardigrada	-	-	-	-
13	Araneae/Acari	*Heptathela hangzhouensis*	NC_005924	*Ixodes uriae*	NC_006078
14	Orthoptera/Hemiptera	*Locusta migratoria*	NC_001712	*Philaenus spumarius*	NC_005944
15	Hymenoptera/Coleoptera	*Melipona bicolor*	NC_004529	*Crioceris duodecimpunctata*	NC_003372
16	Lepidoptera/Diptera	*Antheraea pernyi*	NC_004622	*Bactrocera oleae*	NC_005333
17	Copepoda/Ostracoda	*Tigriopus japonicus*	NC_003979	*Vargula hilgendorfii*	NC_005306
18	Eucarida/Peracarida	*Marsupenaeus japonicus*	NC_007010	*Talitrus saltator*	AY639937
19	Brachiopoda/Phoroniformea	*Terebratalia transversa*	NC_003086	*Phoronis psammophila*	AY368231
20	Acanthocephala/Rotifera	-	-	-	-
21	Aplacophora/Caudofoveata	-	-	-	-
22	Bivalvia/Polyplacophora	*Mytilus galloprovincialis*	NC_006886	*Katharina tunicata*	NC_001636
23	Cephalopoda/Scaphopoda	*Todarodes pacificus*	NC_006354	*Graptacme eborea*	NC_006162
24	Opisthobranchia/Pulmonata	*Aplysia californica*	NC_005827	*Albinaria caerulea*	NC_001761
25	Polychaeta/Oligochaeta	*Clymenella torquata*	NC_006321	*Lumbricus terrestris*	NC_001673
26	Petromyzontidae/Urochordata	-	-	-	-
27	Echinoidea/Asteroidea	-	-	-	-
28	Priapulida/Nematoda	-	-	-	-
29	Cestoda/Trematoda	*Taenia asiatica*	AF445798	*Paragonimus westermani*	NC_002354

COI (previous) COIII (next) Legend

(f) : COIII

	Pairs (Taxon 1/Taxon 2)	Taxon 1 Species name	Taxon 1 Acc. No.	Taxon 2 Species name	Taxon 2 Acc. No.
1	Acoela/Orthonectida	-	-	-	-
2	Turbellaria/Trematoda	-	-	-	-
3	Cestoda/Monogenea	-	-	-	-
4	Urochordata/Cephalochordata	*Doliolum nationalis*	NC_006627	*Epigonichthys lucayanus*	AB110092
5	Chondrichthyes/Petromyzontidae	*Scyliorhinus canicula*	NC_001950	*Petromyzon marinus*	NC_001626
6	Serpentes/Lepidosauria	*Leptotyphlops dulcis*	NC_005961	*Teratoscincus keyserlingii*	NC_007008
7	Echinoidea/Holothuroidea	*Arbacia lixula*	NC_001770	*Cucumaria miniata*	NC_005929
8	Asteroidea/Ophiuroidea	*Pisaster ochraceus*	NC_004610	*Ophiura lutkeni*	NC_005930
9	Enteropneusta/Pterobranchia	-	-	-	-
10	Priapulida/Kinorhyncha	-	-	-	-
11	Nematomorpha/Nematoda	-	-	-	-
12	Onychophora/Tardigrada	-	-	-	-
13	Araneae/Acari	*Heptathela hangzhouensis*	NC_005924	*Ixodes uriae*	NC_006078
14	Orthoptera/Hemiptera	*Locusta migratoria*	NC_001712	*Philaenus spumarius*	NC_005944
15	Hymenoptera/Coleoptera	*Melipona bicolor*	NC_004529	*Crioceris duodecimpunctata*	NC_003372
16	Lepidoptera/Diptera	*Antheraea pernyi*	NC_004622	*Bactrocera oleae*	NC_005333
17	Copepoda/Ostracoda	*Tigriopus japonicus*	NC_003979	*Vargula hilgendorfii*	NC_005306
18	Eucarida/Peracarida	*Marsupenaeus japonicus*	NC_007010	*Parhyale hawaiiensis*	AY639937
19	Brachiopoda/Phoroniformea	*Terebratalia transversa*	NC_003086	*Phoronis psammophila*	AY368231
20	Acanthocephala/Rotifera	-	-	-	-
21	Aplacophora/Caudofoveata	-	-	-	-
22	Bivalvia/Polyplacophora	*Mytilus galloprovincialis*	NC_006886	*Katharina tunicata*	NC_001636
23	Cephalopoda/Scaphopoda	*Todarodes pacificus*	NC_006354	*Graptacme eborea*	NC_006162
24	Opisthobranchia/Pulmonata	*Aplysia californica*	NC_005827	*Albinaria caerulea*	NC_001761
25	Polychaeta/Oligochaeta	*Clymenella torquata*	NC_006321	*Lumbricus terrestris*	NC_001673
26	Petromyzontidae/Urochordata	-	-	-	-
27	Echinoidea/Asteroidea	-	-	-	-
28	Priapulida/Nematoda	-	-	-	-
29	Cestoda/Trematoda	*Taenia asiatica*	AF445798	*Paragonimus westermani*	NC_002354

COII (previous) cytB (next) Legend

(g) : cytB

	Pairs (Taxon 1/Taxon 2)	Taxon 1		Taxon 2	
		Species name	Acc. No.	Species name	Acc. No.
1	Acoela/Orthonectida	-	-	-	-
2	Turbellaria/Trematoda	-	-	-	-
3	Cestoda/Monogenea	-	-	-	-
4	Urochordata/Cephalochordata	*Doliolum nationalis*	NC_006627	*Epigonichthys lucayanus*	AB110092
5	Chondrichthyes/Petromyzontidae	*Scyliorhinus canicula*	NC_001950	*Petromyzon marinus*	NC_001626
6	Serpentes/Lepidosauria	*Leptotyphlops dulcis*	NC_005961	*Teratoscincus keyserlingii*	NC_007008
7	Echinoidea/Holothuroidea	*Arbacia lixula*	NC_001770	*Cucumaria miniata*	NC_005929
8	Asteroidea/Ophiuroidea	*Pisaster ochraceus*	NC_004610	*Ophiura lutkeni*	NC_005930
9	Enteropneusta/Pterobranchia	-	-	-	-
10	Priapulida/Kinorhyncha	-	-	-	-
11	Nematomorpha/Nematoda	-	-	-	-
12	Onychophora/Tardigrada	-	-	-	-
13	Araneae/Acari	*Heptathela hangzhouensis*	NC_005924	*Ixodes uriae*	NC_006078
14	Orthoptera/Hemiptera	*Locusta migratoria*	NC_001712	*Philaenus spumarius*	NC_005944
15	Hymenoptera/Coleoptera	*Melipona bicolor*	NC_004529	*Crioceris duodecimpunctata*	NC_003372
16	Lepidoptera/Diptera	*Antheraea pernyi*	NC_004622	*Bactrocera oleae*	NC_005333
17	Copepoda/Ostracoda	*Tigriopus japonicus*	NC_003979	*Vargula hilgendorfii*	NC_005306
18	Eucarida/Peracarida	*Marsupenaeus japonicus*	NC_007010	*Parhyale hawaiiensis*	AY639937
19	Brachiopoda/Phoroniformea	*Terebratalia transversa*	NC_003086	*Phoronis psammophila*	AY368231
20	Acanthocephala/Rotifera	-	-	-	-
21	Aplacophora/Caudofoveata	-	-	-	-
22	Bivalvia/Polyplacophora	*Mytilus galloprovincialis*	NC_006886	*Katharina tunicata*	NC_001636
23	Cephalopoda/Scaphopoda	*Todarodes pacificus*	NC_006354	*Graptacme eborea*	NC_006162
24	Opisthobranchia/Pulmonata	*Aplysia californica*	NC_005827	*Albinaria caerulea*	NC_001761
25	Polychaeta/Oligochaeta	*Clymenella torquata*	NC_006321	*Lumbricus terrestris*	NC_001673
26	Petromyzontidae/Urochordata	-	-	-	-
27	Echinoidea/Asteroidea	-	-	-	-
28	Priapulida/Nematoda	-	-	-	-
29	Cestoda/Trematoda	-	-	-	-

COIII (previous)   NADH1 (previous)   Legend

(h) : NADH1

Pairs (Taxon 1/Taxon 2)		Taxon 1 Species name	Taxon 1 Acc. No.	Taxon 2 Species name	Taxon 2 Acc. No.
1	Acoela/Orthonectida	-	-	-	-
2	Turbellaria/Trematoda	-	-	-	-
3	Cestoda/Monogenea	-	-	-	-
4	Urochordata/Cephalochordata	*Doliolum nationalis*	NC_006627	*Epigonichthys lucayanus*	AB110092
5	Chondrichthyes/Petromyzontidae	*Scyliorhinus canicula*	NC_001950	*Petromyzon marinus*	NC_001626
6	Serpentes/Lepidosauria	*Leptotyphlops dulcis*	NC_005961	*Teratoscincus keyserlingii*	NC_007008
7	Echinoidea/Holothuroidea	*Arbacia lixula*	NC_001770	*Cucumaria miniata*	NC_005929
8	Asteroidea/Ophiuroidea	*Pisaster ochraceus*	NC_004610	*Ophiura lutkeni*	NC_005930
9	Enteropneusta/Pterobranchia	-	-	-	-
10	Priapulida/Kinorhyncha	-	-	-	-
11	Nematomorpha/Nematoda	-	-	-	-
12	Onychophora/Tardigrada	-	-	-	-
13	Araneae/Acari	*Heptathela hangzhouensis*	NC_005924	*Ixodes uriae*	NC_006078
14	Orthoptera/Hemiptera	*Locusta migratoria*	NC_001712	*Philaenus spumarius*	NC_005944
15	Hymenoptera/Coleoptera	*Melipona bicolor*	NC_004529	*Crioceris duodecimpunctata*	NC_003372
16	Lepidoptera/Diptera	*Antheraea pernyi*	NC_004622	*Bactrocera oleae*	NC_005333
17	Copepoda/Ostracoda	*Tigriopus japonicus*	NC_003979	*Vargula hilgendorfii*	NC_005306
18	Eucarida/Peracarida	*Marsupenaeus japonicus*	NC_007010	*Parhyale hawaiiensis*	AY639937
19	Brachiopoda/Phoroniformea	*Terebratalia transversa*	NC_003086	*Phoronis psammophila*	AY368231
20	Acanthocephala/Rotifera	-	-	-	-
21	Aplacophora/Caudofoveata	-	-	-	-
22	Bivalvia/Polyplacophora	*Mytilus galloprovincialis*	NC_006886	*Katharina tunicata*	NC_001636
23	Cephalopoda/Scaphopoda	*Todarodes pacificus*	NC_006354	*Graptacme eborea*	NC_006162
24	Opisthobranchia/Pulmonata	*Aplysia californica*	NC_005827	*Albinaria caerulea*	NC_001761
25	Polychaeta/Oligochaeta	*Clymenella torquata*	NC_006321	*Lumbricus terrestris*	NC_001673
26	Petromyzontidae/Urochordata	-	-	-	-
27	Echinoidea/Asteroidea	-	-	-	-
28	Priapulida/Nematoda	-	-	-	-
29	Cestoda/Trematoda	*Taenia asiatica*	AF445798	*Paragonimus westermani*	NC_002354

cytB (previous)   NADH4 (next)   Legend

(i) : NADH4

	Pairs (Taxon 1/Taxon 2)	Taxon 1		Taxon 2	
		Species name	Acc. No.	Species name	Acc. No.
1	Acoela/Orthonectida	-	-	-	-
2	Turbellaria/Trematoda	-	-	-	-
3	Cestoda/Monogenea	-	-	-	-
4	Urochordata/Cephalochordata	*Doliolum nationalis*	NC_006627	*Epigonichthys lucayanus*	AB110092
5	Chondrichthyes/Petromyzontidae	*Scyliorhinus canicula*	NC_001950	*Petromyzon marinus*	NC_001626
6	Serpentes/Lepidosauria	*Leptotyphlops dulcis*	NC_005961	*Teratoscincus keyserlingii*	NC_007008
7	Echinoidea/Holothuroidea	*Arbacia lixula*	NC_001770	*Cucumaria miniata*	NC_005929
8	Asteroidea/Ophiuroidea	*Pisaster ochraceus*	NC_004610	*Ophiura lutkeni*	NC_005930
9	Enteropneusta/Pterobranchia	-	-	-	-
10	Priapulida/Kinorhyncha	-	-	-	-
11	Nematomorpha/Nematoda	-	-	-	-
12	Onychophora/Tardigrada	-	-	-	-
13	Araneae/Acari	*Heptathela hangzhouensis*	NC_005924	*Ixodes uriae*	NC_006078
14	Orthoptera/Hemiptera	*Locusta migratoria*	NC_001712	*Philaenus spumarius*	NC_005944
15	Hymenoptera/Coleoptera	*Melipona bicolor*	NC_004529	*Crioceris duodecimpunctata*	NC_003372
16	Lepidoptera/Diptera	*Antheraea pernyi*	NC_004622	*Bactrocera oleae*	NC_005333
17	Copepoda/Ostracoda	*Tigriopus japonicus*	NC_003979	*Vargula hilgendorfii*	NC_005306
18	Eucarida/Peracarida	*Marsupenaeus japonicus*	NC_007010	*Parhyale hawaiiensis*	AY639937
19	Brachiopoda/Phoroniformea	*Terebratalia transversa*	NC_003086	*Phoronis psammophila*	AY368231
20	Acanthocephala/Rotifera	-	-	-	-
21	Aplacophora/Caudofoveata	-	-	-	-
22	Bivalvia/Polyplacophora	*Mytilus galloprovincialis*	NC_006886	*Katharina tunicata*	NC_001636
23	Cephalopoda/Scaphopoda	*Todarodes pacificus*	NC_006354	*Graptacme eborea*	NC_006162
24	Opisthobranchia/Pulmonata	*Aplysia californica*	NC_005827	*Albinaria caerulea*	NC_001761
25	Polychaeta/Oligochaeta	*Clymenella torquata*	NC_006321	*Lumbricus terrestris*	NC_001673
26	Petromyzontidae/Urochordata	-	-	-	-
27	Echinoidea/Asteroidea	-	-	-	-
28	Priapulida/Nematoda	-	-	-	-
29	Cestoda/Trematoda	*Taenia asiatica*	AF445798	*Paragonimus westermani*	NC_002354

NADH1 (previous)   Legend
