# Supplementary material for: The influence of body size and net diversification rate on molecular evolution during the radiation of animal phyla
Source: BMC Evol Biol. 2007 Jun 26;7:95. doi: 10.1186/1471-2148-7-95 (PMC1929056; doi:10.1186/1471-2148-7-95)
Supplement: Additional file 8 — Alignment length before and after removing unalignable sequence. Bases were excised manually in Bioedit [69]. [file 1471-2148-7-95-S8.pdf]

	Alignment length BEFORE removing un-alignable part	Alignment length AFTER removing un-alignable part	% removed
**18S**	2567	1313	48.9
**28S**	5913	1403	76.3
**ef1a**	2358	1088	53.9
**COI**	1711	824	51.8
**COII**	769	611	20.5
**COIII**	952	371	61.0
**cytB**	1243	635	48.9
**NADH1**	1034	314	69.6
**NADH4**	1469	413	71.9

mean=55.87
