# Supplementary material for: The influence of body size and net diversification rate on molecular evolution during the radiation of animal phyla
Source: BMC Evol Biol. 2007 Jun 26;7:95. doi: 10.1186/1471-2148-7-95 (PMC1929056; doi:10.1186/1471-2148-7-95)
Supplement: Additional file 9 — Comparison pairs used for each gene. Each pair used is indicated by an 'X'. Also shown are the total number of comparison pairs used, and the alignment lengths in base pairs. [file 1471-2148-7-95-S9.pdf]

		Nuclear genes			Mitochondrial genes						Concatenations	
		18S	28S	ef1a	COI	COII	COIII	cytb	NADH1	NADH4	C100	C20
	Number of comparison pairs used	25	23	14	20	17	17	16	17	17	20	16
	Alignment length	1311	1399	1086	822	609	369	633	312	411	3156	3156
	Comparison pairs used :											
1	Acoela/Orthonectida	x										
2	Turbellaria/Trematoda	x	x	x	x						x	
3	Cestoda/Monogenea	x	x	x	x						x	
4	Urochordata/Cephalochordata	x	x		x	x	x	x	x	x	x	x
5	Chondrichthyes/Petromyzontidae	x	x		x	x	x	x	x	x	x	x
6	Serpentes/Lepidosauria	x			x	x	x	x	x	x	x	x
7	Echinoidea/Holothuroidea	x	x		x	x	x	x	x	x	x	x
8	Asteroidea/Ophiuroidea	x	x		x	x	x	x	x	x	x	x
9	Enteropneusta/Pterobranchia	x	x									
10	Priapulida/Kinorhyncha	x	x									
11	Nematomorpha/Nematoda	x	x									
12	Onychophora/Tardigrada	x	x	x								
13	Araneae/Acari	x	x	x	x	x	x	x	x	x	x	x
14	Orthoptera/Hemiptera	x	x	x	x	x	x	x	x	x	x	x
15	Hymenoptera/Coleoptera	x	x	x	x	x	x	x	x	x	x	x
16	Lepidoptera/Diptera	x	x	x	x	x	x	x	x	x	x	x
17	Copepoda/Ostracoda	x	x	x	x	x	x	x	x	x	x	x
18	Eucarida/Peracarida	x	x	x	x	x	x	x	x	x	x	x
19	Brachiopoda/Phoroniformea	x	x		x	x	x	x	x	x	x	x
20	Acanthocephala/Rotifera	x	x		x						x	
21	Aplacophora/Caudofoveata	x	x		x						x	
22	Bivalvia/Polyplacophora	x	x	x	x	x	x	x	x	x	x	x
23	Cephalopoda/Scaphopoda	x	x		x	x	x	x	x	x	x	x
24	Opisthobranchia/Pulmonata	x	x		x	x	x	x	x	x	x	x
25	Polychaeta/Oligochaeta	x	x	x	x	x	x	x	x	x	x	x
26	Petromyzontidae/Urochordata			x								
27	Echinoidea/Asteroidea			x								
28	Priapulida/Nematoda			x								
29	Cestoda/Trematoda					x	x		x	x		
